# Effect of Air Exposure of ZnMgO Nanoparticle Electron Transport Layer on Efficiency of Quantum-Dot Light-Emitting Diodes

**DOI:** 10.1021/acsami.1c01898

**Published:** 2021-04-23

**Authors:** Maciej Chrzanowski, Grzegorz Zatryb, Piotr Sitarek, Artur Podhorodecki

**Affiliations:** Department of Experimental Physics, Wroclaw University of Science and Technology, Wybrzeze Wyspianskiego 27, 50-370 Wroclaw, Poland

**Keywords:** quantum dots, light-emitting diodes, ZnMgO nanoparticles, air exposure, charge balance, device stability

## Abstract

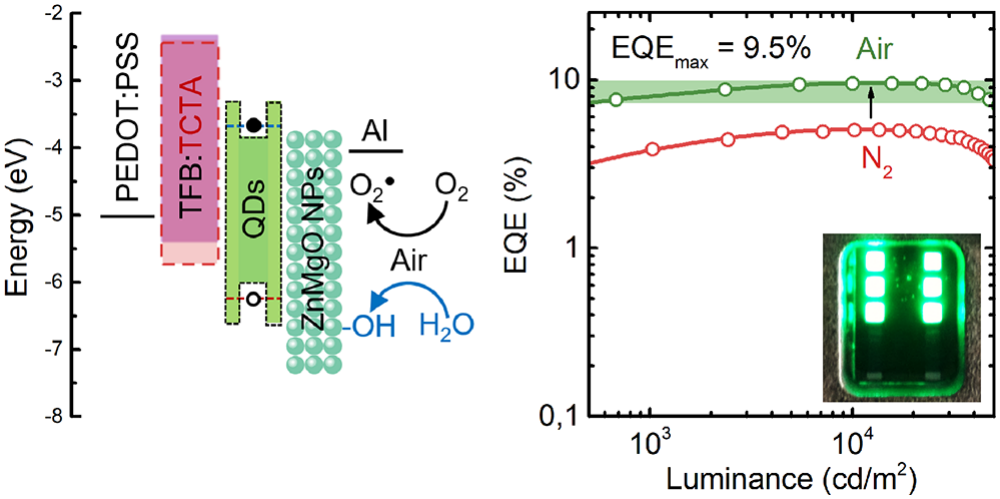

We demonstrate the effect of air exposure on optical and electrical properties of ZnMgO nanoparticles (NPs) typically exploited as an electron transport layer in Cd-based quantum-dot light-emitting diodes (QLEDs). We analyze the roles of air components in modifying the electrical properties of ZnMgO NPs, which reveals that H_2_O enables the reduction of hole leakage while O_2_ alters the character of charge transport due to its ability to trap electrons. As a result, the charge balance in the QDs layer is improved, which is confirmed by voltage-dependent measurements of photoluminescence quantum yield. The maximum external quantum efficiency is improved over 2-fold and reaches the value of 9.5% at a luminance of 10^4^ cd/m^2^. In addition, we investigate the problem of electron leakage into the hole transport layer and show that trap-mediated electron transport in the ZnMgO layer caused by adsorbed O_2_ ensures a higher leakage threshold. This work also provides an insight into the possible disadvantages of device contact with air as well as problems and challenges that might occur during open-air fabrication of QLEDs.

## Introduction

Colloidal quantum-dot light-emitting diodes (QLEDs) have attracted considerable attention in the optoelectronic industry due to their remarkable performance, which makes them promising candidates for displays and lightning technology with a particular emphasis on color purity, brightness, and emission tunability.^1,2^ Although state-of-the-art devices have reached excellent performance in terms of both efficiency and stability,^3,4^ some papers reported abnormal behavior during device operation and storage including positive aging,^5^ instabilities of current and luminance during device testing,^6^ and hole transport layer (HTL) degradation.^7^

Recently, a lot of attention has also been paid to encapsulation as a potential source of unwanted chemical moieties such as water or organic acids.^8^ For instance, it has been proposed that contamination with water, which is created by chemical reaction between the resin and ZnMgO electron transport layer (ETL), is a key factor influencing device lifetime.^9−11^ Some authors have pointed to spontaneous interfacial reaction of ZnMgO with Al as a major cause of the aging process,^12^ while others proposed vacancy reduction in ZnMgO as a possible reason.^13^ Recently, a more sophisticated phenomenon such as resistive switching, i.e., electric-field-induced oxygen vacancies migration, which is an intrinsic property of ZnMgO, has been investigated as potentially detrimental to operational stability.^14^

From the point of view of scalable QLED manufacturing, open-air fabrication of QLED is highly desired. For this reason, environmental testing is an important part of research on optoelectronic devices. QLEDs are typically fabricated in a chemically inert gas atmosphere (N_2_ or Ar), but recently, the influence of air exposition on properties of ZnO nanoparticles (NPs) has started to be the scope of intense scrutiny. For instance, the water adsorbed in humid air has been proposed as a key to understanding the process of QLEDs aging.^15^ Particularly, it was shown that H_2_O reduces the number of active sites at the ZnO surface, which results in reduced hole leakage in the device.

In this paper, we illustrate the impact of short air exposure on QLED performance and attempt to describe how water and oxygen are modifying the electrical transport of the ZnMgO layer. We also investigate the optical properties of QDs and ZnMgO NPs and interpret device operation in terms of charge balance in the QDs layer. We also analyze the problem of electron leakage in HTL. Finally, we perform stability tests and discuss the limitations of open-air fabrication of QLEDs.

## Results and Discussion

### QLED Characteristics

**Figure 1:**
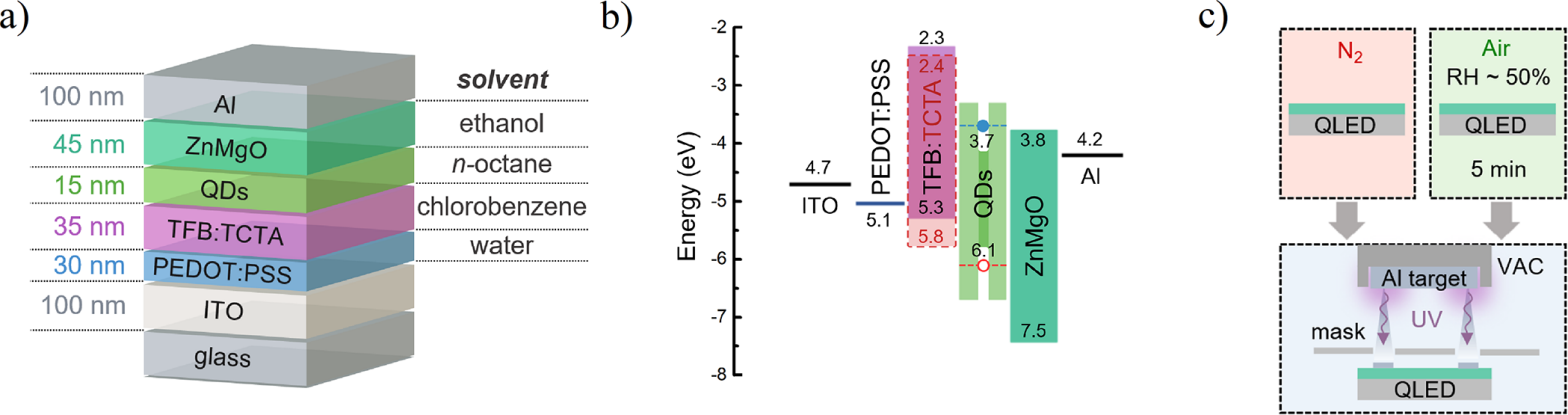
(a) Structure and (b) band diagram of QLED under investigation. (c) Scheme showing the difference in the ZnMgO treatment.

The device under investigation has a standard structure: ITO/PEDOT:PSS (30 nm)/TFB:TCTA (35 nm)/QDs (15 nm)/ZnMgO (45 nm)/Al (Figure 1a). This configuration has been intensively studied, and it showed the highest performance in terms of low turn-on voltage and high brightness for blue, green, and red Cd-based and InP QDs.^16−20^ These features are attributed to the excellent mobility of TFB (1 × 10^–2^ cm^2^/(V s)) compared to other polymers or small molecules as well as efficient hole injection in PEDOT:PSS/TFB arising from Fermi level pinning.^21^ However, the external quantum efficiency (EQE) of Cd-based QLEDs is vulnerable to offset between the highest occupied molecular orbital (HOMO) level of TFB and QDs hole level which is particularly high for ZnS-coated QDs.^22^ As a result, device performance is very sensitive to the electron accumulation in the QDs layer that leads to a drop of radiative recombination efficiency as a result of Auger processes.^23^ Accumulated electrons tend also to leak into the adjacent TFB layer and cause electrochemical reactions deteriorating hole transport in TFB which is essential for device stability.^24^

For this reason, it is important to provide a strategy either to minimize the hole injection barrier or to reduce electron injection.^25^ In this paper, we show that the second approach can be done by modifying electrical transport in ZnMgO NPs by air exposition. In our approach, we incorporated CdSe@ZnS/ZnS QDs emitting at 515 nm (FWHM ∼ 21 nm, PLQY ∼ 55%) with a thickness of a single monolayer into QLED, which assures optimal device performance in terms of brightness and turn-on voltage (Figure S1a,b). As an ETL, we used alloyed ZnMgO NPs (15 mol % Mg) which have been recently proposed to balance charge injection into QDs by suppressing electron current.^26,27^ They have also been proven effective in reducing the QDs photoluminescence (PL) quenching which arises from hole trapping by defect states such as oxygen vacancies in standard ZnO NPs.^11,12,28^ To alleviate the problem of high injection barrier at TFB/QDs interface, we also adopted an approach based on HTL composed of TFB mixed with small-molecule material TCTA.^29^ Although hole mobility in TCTA (3 × 10^–4^ cm^2^/(V s)) is smaller than in TFB, its HOMO level is laying deeper than that of TFB (Figure 1b),^30^ and therefore TFB:TCTA HTL mitigates hole injection into QDs. Enhanced hole injection improves charge balance, which is evidenced by the increase of EQE from 3.5% to 4.5% and over a 2-fold increase of luminance (Figure S2a,b).

Using this QLED structure, we performed environmental tests by exposing the device to humid air. The ohmic region is a distinct feature of the *J*–*V* characteristics of QLEDs because it can reflect the transport properties of ETL processed in different conditions. More specifically, the amount of gas adsorbed by the ZnMgO layer depends on the sequence of device processing (Figure S3a). For a device exposed to the air before and after Al deposition (denoted as Air/Air), the current in the ohmic region is the lowest, but it rises if the latter stage is missing (Air/N_2_). Conversely, if the ETL is not exposed to air before Al deposition (N_2_/Air), it has a smaller chance to adsorb gases because the Al layer impedes air diffusion, which translates to a higher current density. Gas adsorption is, however, still possible because the ohmic current of the device prepared in N_2_ is decreasing after exposition to air within 5–10 min (Figure S3b).

**Figure 2:**
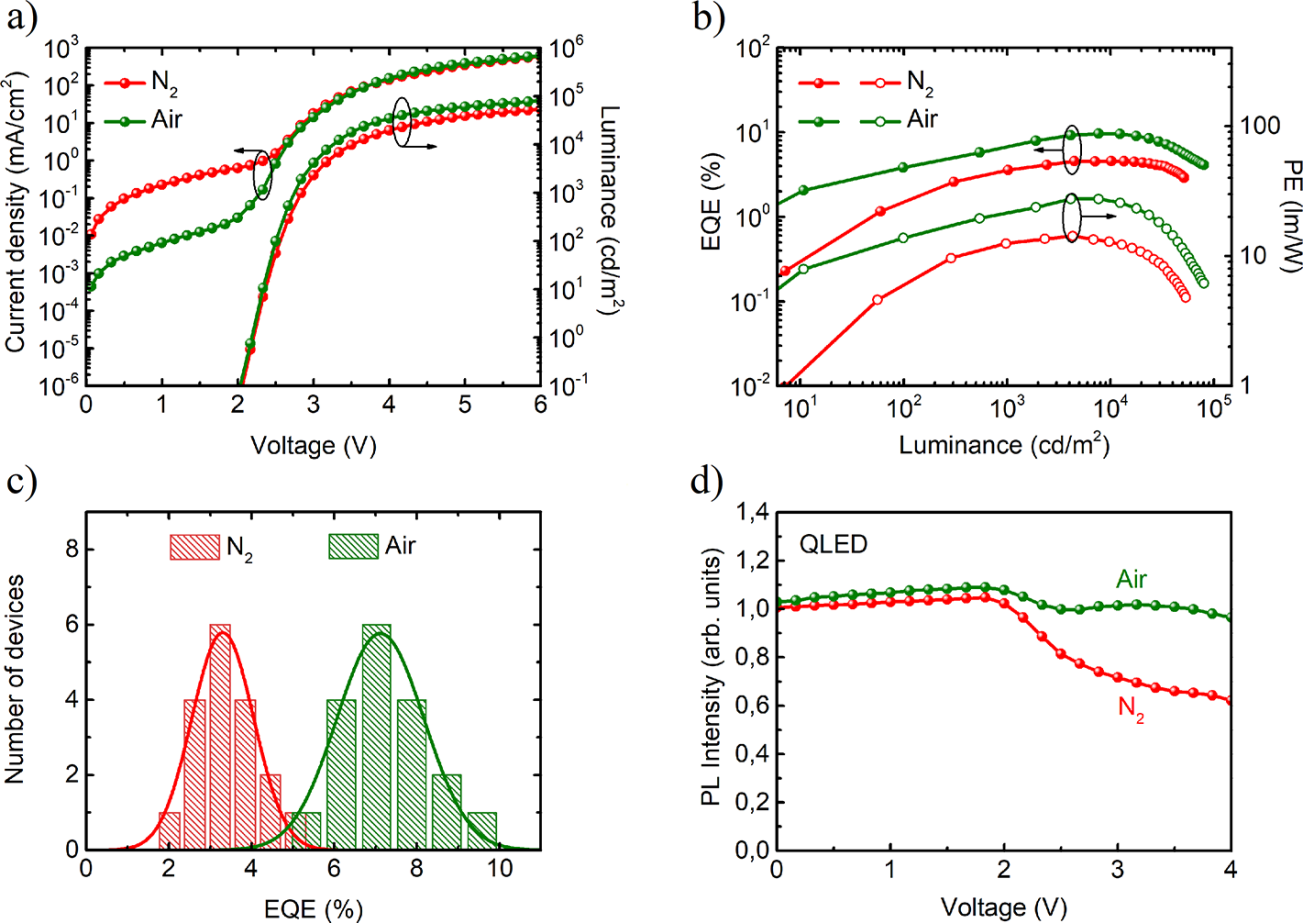
(a) *J*–*V*–*L* and (b) EQE–*J* characteristics of QLEDs with ZnMgO ETL exposed for 5–10 min to humid air (Air) or processed under inert atmosphere (N_2_). (c) EQE statistics for corresponding devices. (d) Voltage-dependent PL intensity measured from QLED pixels.

For simplicity, in this paper, we analyzed the performances of two devices: one produced completely in N_2_ and the other exposed to air before Al deposition (Figure 1c). EQE measured for each device reflects the amount of adsorbed gas because the latter device is characterized by over 2-fold better performance both in terms of EQE and luminance (Figure 2a,b). The average EQE of the optimized QLED with TFB:TCTA (50 wt %) is estimated at 7.1% based on the statistics shown in Figure 2c, and the maximum device EQE is 9.5%, which corresponds to a power efficiency of 30 lm/W and a current efficiency of 30 cd/A.

### Chemical Analysis of ZnMgO NPs

To explain the observed effects, we first examined the optical properties of QDs embedded in the device. For this aim, PL decays were measured from pixel areas (Figure S4a). One would expect that PL lifetime should be different in both samples due to a population of defect states in the ZnMgO layer by electrons from Al contact that is likely to be responsible for trap-assisted carrier recombination as speculated previously.^12^ The number of active adsorbing sites for O_2_ in ZnMgO is significantly reduced after exposing the sample to air because these traps become terminated with −OH groups coming from chemisorbed H_2_O molecules.^15^ Consequently, after air contact, the nonradiative recombination channel should be blocked in a similar manner as inserting the PVK blocking layer (5 nm) increases PL lifetime from 4.6 to 5.2 ns (Figure S4b). However, PL lifetimes turned out to be the same for both QLEDs (∼4.5 ns), which suggests that the adsorbed gases do not contribute to QDs PL quenching. In our case, the reaction of resin and ZnMgO and contact metallization by a spontaneous reaction between Al and ZnMgO have been excluded as factors that could distort measurements because both QDs PL and the current density of the encapsulated device were stable during device storage (Figure S5a,b).

Because optical properties of QDs were preserved irrespectively of ZnMgO treatment, we have focused our attention on charge balance in the QDs layer, which should be strongly related to EQE, provided that the electrical transport is suspected to be altered after the contact of ZnMgO with air. For this aim, we measured the voltage-dependent photoluminescence quantum yield (PLQY) (see the Experimental Section). For the device exposed to air, PLQY is increased by 40% at a voltage corresponding to EQE maximum (∼3.3 V), which implies that the QDs layer remains more neutral in the charge recombination regime (Figure 2d). This confirms that EQE improvement is related to more balanced carrier injection which evidently must be the result of different transport properties of the ZnMgO layer.

**Figure 3:**
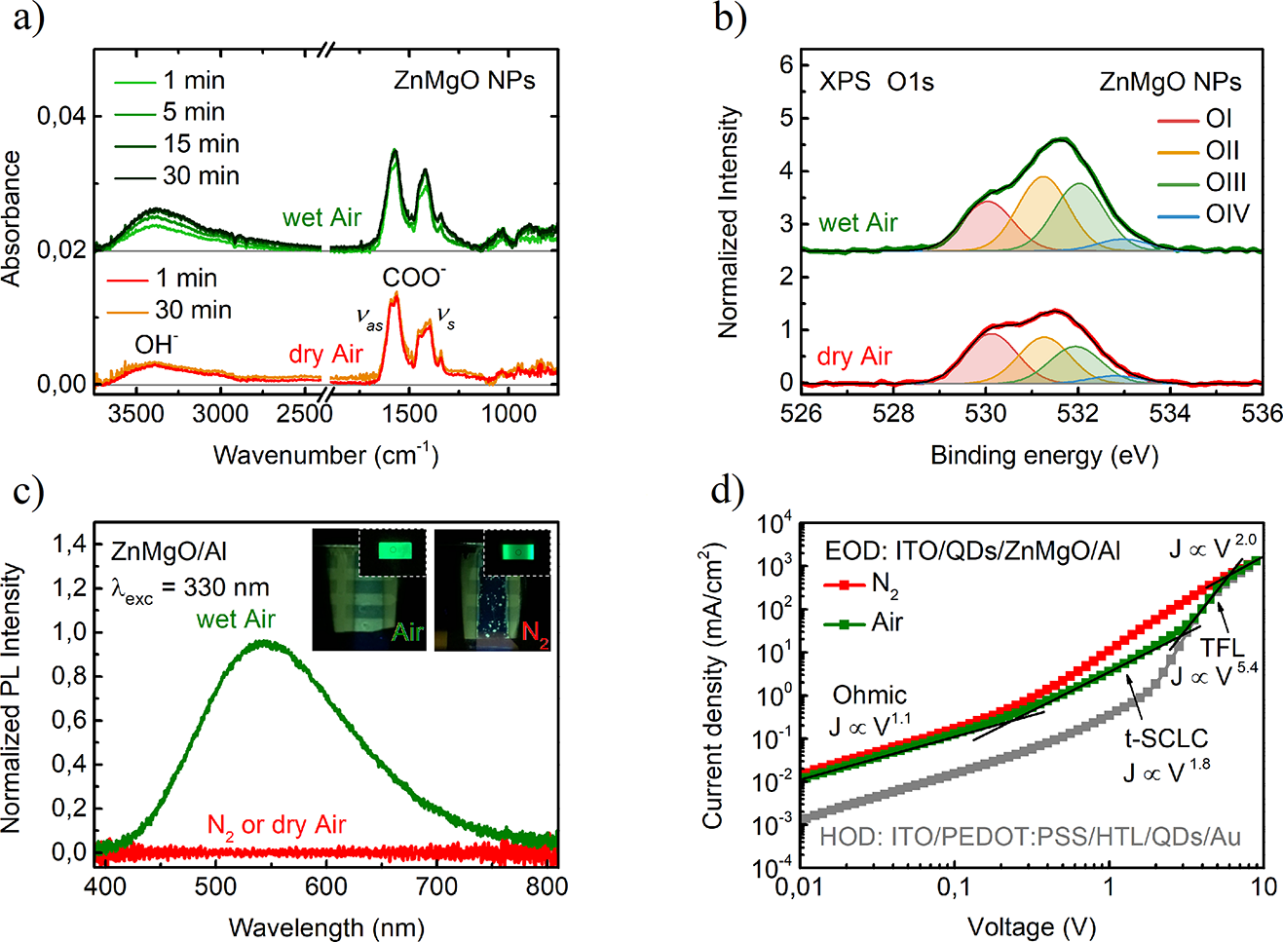
(a) FTIR spectra of ZnMgO NPs exposed to dry or wet air. (b) XPS spectra of ZnMgO NPs treated with dry or wet air. (c) PL spectra of ZnMgO NPs with sputtered Al layer. The inset shows photos of ZnMgO/Al structures exposed to wet air under UV illumination and electroluminescence of corresponding QLEDs. (d) *J*–*V* characteristics of hole-only device (HOD) and electron-only devices (EODs).

One important factor limiting the performance of QLEDs with ZnMgO ETL is a hole leakage,^31^ which arises from trap-mediated transport.^10−12^ It is known that H_2_O molecules passivate active sites in ZnMgO NPs, and therefore it is apparent that the difference between EQEs of devices produced in N_2_ results from reduced hole leakage current.^14^ Our ZnMgO NPs are fabricated in the inert gas atmosphere (N_2_) and are capped with acetic acid ligands as evidenced by symmetric (ν_s_) and asymmetric (ν_as_) stretching COO^–^ bands in Fourier transform infrared (FTIR) spectra (Figure 3a). The FTIR spectra measured before and after wet air treatment reveal that the number of OH groups is increased, which confirms that water terminates some of the active sites at the NPs surface. The changes observed in FTIR correlate well with a decrease of the ohmic current experiment (Figure S3b). An exposition time longer than 10 min has a negligible effect on current density and FTIR spectra, and therefore 5–10 min in wet air is enough to improve device performance. However, ZnMgO treatment in dry air (RH ∼ 30%) is insufficient to modify NPs surface and consequently cannot guarantee the improvement of device performance. In contrast, at a humidity level above 60% water vapor condensates on the surface of ZnMgO film and soaks into subjacent layers, which quickly deteriorates layer uniformity and ruins device performance.

Further chemical composition analysis was performed by X-ray photoelectron spectroscopy (XPS). The acquired O 1s spectra shown in Figure 3b are deconvoluted with Gaussian peaks corresponding to metal oxide (O_I_, 530.1 eV) and oxygen-deficient oxide (O_II_, 531.3 eV) and two peaks assigned to loosely bounded oxygen in the form of hydroxide (O_III_, 532.0 eV) and chemisorbed oxygen species (O_IV_, 532.9 eV).^28,32^ The area ratios of peaks O_II_, O_III_, and O_IV_ to the O_I_ signal are increased by 62%, 83%, and 51%, which not only confirms the formation of hydroxide after wet air treatment but also suggests an increased number of oxygen vacancies and adsorbed oxygen in the form of O_2_ or H_2_O. We also noticed that the ZnMgO layer is easily dissolved by the native solvent (ethanol) in N_2_ or in dry air, but the solubility is irreversibly lost if the sample had spent enough time in humid air (RH ∼ 50%), which confirms reconfiguration of surface sites by adsorbed H_2_O. It is important to note that hole leakage current and charge balance are correlated because higher hole concentration means more balanced charge distribution in QDs as well as higher recombination rate which is reflected by improved luminance. However, as will be shown below, these are not the only factors limiting device performance because air exposition also changes the electron transport.

### Optical Properties of ZnMgO NPs

Before we investigated the conductivity of ZnMgO NPs, we studied their optical properties to take an insight into the process of gas adsorption. As shown recently, visible emission in ZnO NPs originates from physisorbed O_2_ molecules rather than oxygen vacancies.^32^ Oxygen is capable to trap excited electrons under UV excitation forming emissive superoxide (O_2_^–^) which quenches near band edge PL, while other charge transfer states such as those formed by N_2_ remain weakly emissive.^33^

Our ZnMgO NPs exhibit weak green PL under excitation near the absorption edge (330 nm) in N_2_ which is, however, completely quenched after Al deposition (Figure 3c). PL disappears even if the sample is exposed to dry air (RH < 30%), where both O_2_ and N_2_ molecules are expected to be adsorbed. It turns out that gas adsorbates are evacuated under vacuum during Al sputtering within several seconds due to strong UV radiation coming from Ar plasma discharge glow. This is confirmed by independent tests in a vacuum chamber where visible PL is quenched within <1 min after the sample is excited with UV light (330 nm) although this process is probably faster during Al sputtering due to the higher intensity of plasma glow (Figure S6). The green emission of ZnMgO NPs rebuilds instantly after the chamber is vented with air, but it is also detected under pure N_2_, which might be connected to some O_2_ contamination.

In contrast to the above experiments, the ZnMgO layer exposed to humid air (RH ∼ 50%) shows green emission even after Al sputtering (Figure 3c). We suggest that adsorbed H_2_O causes a temporal morphological change in the packing density of NPs and enables oxygen to be captured in the pores of the layer. This is consistent with the fact that the PL of ZnMgO NPs treated with humid air is stable much longer under vacuum, which should also be the case during the sputtering process. However, the PL of ZnMgO still can be completely quenched by UV light, which proves that adsorbed O_2_ molecules rather than oxygen vacancies are the primary source of visible emission. The interplay of water and oxygen is also clearly visible after Al deposition. The device exposed to dry air experiences O_2_ adsorption only outside the pixel area. However, in humid air, O_2_ can also penetrate under the Al layer within a depth of a few hundred micrometers, which causes electroluminescence (EL) improvement at pixel edges. Although this effect confirms the role of H_2_O in introducing O_2_ into the ZnMgO layer, it might become a serious obstacle during open-air fabrication of QLEDs devices without the precise control of air humidity.

### Electrical Properties of ZnMgO ETL

Gas adsorbates are known to modify electric conductivity in nanocrystalline ZnO in a different way.^32^ Unlike nitrogen, oxygen with its ability to trap electrons is responsible for reduced ZnO conductivity. In contrast, H_2_O can dissociate into H^+^ and OH^–^ ions in contact with ZnO and therefore permanently stabilize surface active sites by the formation of Zn–OH. Bounded hydrogen, which acts as an electron donor, is suggested to be responsible for enhanced n-type conductivity.

Oxygen has the most pronounced impact on electrical transport because it acts as an electron scavenger, which is confirmed by *J*–*V* measurements of electron-only devices (EODs) with the structure of ITO/QDs/ZnMgO/Al with ZnMgO either exposed to air or prevented from air access (Figure 3d). The *J*–*V* characteristics exhibit four different current regimes: ohmic current, trap-limited space charge limited current (t-SCLC), trap-filled limited (TFL) current, and space charge limited current (SCL).^34^ The current densities in ohmic and SCL regimes are comparable, which means that Al deposition does not create any shortcuts and Schottky barriers at the Al/ZnMgO interface are not altered. However, the TFL regime for ZnMgO exposed to air is characterized by a higher power exponent and higher threshold voltage (*V*_TFL_), which indicates the presence of additional trap states introduced by oxygen. Electron current in QLED exposed to air is therefore expected to be trap-limited in the low voltage range. The comparison of EOD and hole-only device (HOD) characteristics in Figure 3d shows that the mismatch between electron and hole injection is smaller for EOD exposed to air, which supports the claim that, apart from blocked hole leakage, reduced electron injection is the second factor improving the charge balance in the QDs layer.

To analyze device operation more thoroughly, we analyzed optical properties of the TFB layer because HTLs with high electron mobility such as TFB are vulnerable to electron overflow.^35^ For instance, for QLED with discontinuous QDs layer (denoted as <1 ML), EL intensity is strongly reduced, which results in more than a 2-fold drop of EQE (see Figure S1b). This is explained by electron leakage into HTL and is confirmed by parasitic TFB signal emerging at a short-wavelength shoulder of the QDs PL spectrum (Figure S7a,b), which is however quickly quenched as a result of electrochemical reduction of TFB.^24^ In QLED with 1 ML thick QDs layer, this effect is still severe, and after the voltage is increased above ∼3.4 V, the HTL signal disappears irreversibly (Figure S8a). That voltage can be considered as a threshold above which electrons start to leak. The threshold voltage is smaller for the device with ZnMgO prevented from air contact as shown in Figure S8b, which implies that trap-limited transport in ZnMgO exposed to air creates also a synergetic effect that prevents undesirable electron leakage. This allows us also to explain the difference in current densities in the high voltage range for QLEDs prepared in different conditions (see Figure 2a). Although the higher current density of the device exposed to air could be ascribed to enhanced electron mobility in ZnMgO as a result of adsorbed H_2_O acting as electron donor,^11^ or enhanced electron injection due to oxidation of Al layer,^36,37^ it is more likely that less intense electron leak into the TFB layer is the primary source of the observed difference.

**Figure 4:**
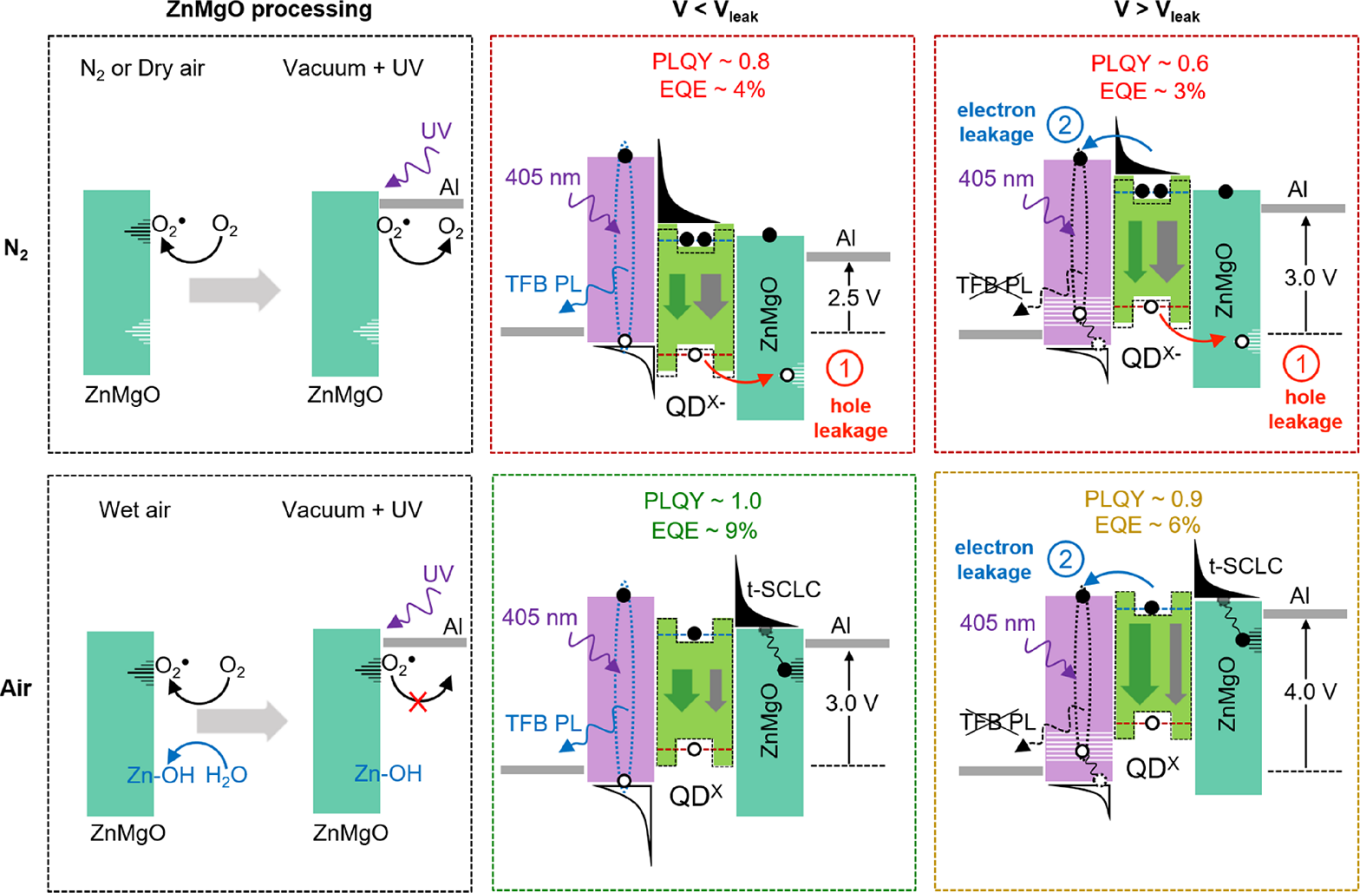
Summary of the effect of ZnMgO treatment on the operation mechanism of QLED below and above electron leakage threshold. The abbreviations PLQY and EQE refer to the relative photoluminescence quantum yield and the external quantum efficiency, respectively.

The scheme in Figure 4 summarizes the impact of both ZnMgO treatment and electron leakage on device performance. If the device is exposed to dry air, oxygen is quickly adsorbed by ZnMgO NPs at the surface of the film. During cathode sputtering, electrons trapped by O_2_ recombine with holes photogenerated by strong UV radiation resulting in O_2_ desorption, and any residual oxygen is eventually consumed by reactive Al. As a result, EQE is poor because EL is quenched by hole leakage mediated by surface trap states in ZnMgO and excessive electron injection which also leads to a low voltage threshold for electron leakage. In wet air, however, H_2_O penetrates the ZnMgO layer and introduces dissolved oxygen into ZnMgO pores. For this reason, not only is hole leakage suppressed by OH groups passivating ZnMgO NPs surface ions, but also electron transport is changed into trap-limited by captured O_2_. The reason for the EQE boost can be understood by the fact that, on the one hand, reduced hole leakage provides a higher charge balance within the QDs layer and, on the other hand, trap-limited electron transport enables to reach the charge balance before electron leak starts. As a result, at a voltage of 3.3 V corresponding to EQE maximum, the recombination zone is completely confined in QDs, which is supported by the fact that TFB PL is preserved. Exposition to wet air is therefore vital for device performance because it blocks hole leakage and suppresses electron injection improving charge balance effectively.

### Device Lifetime

**Figure 5:**
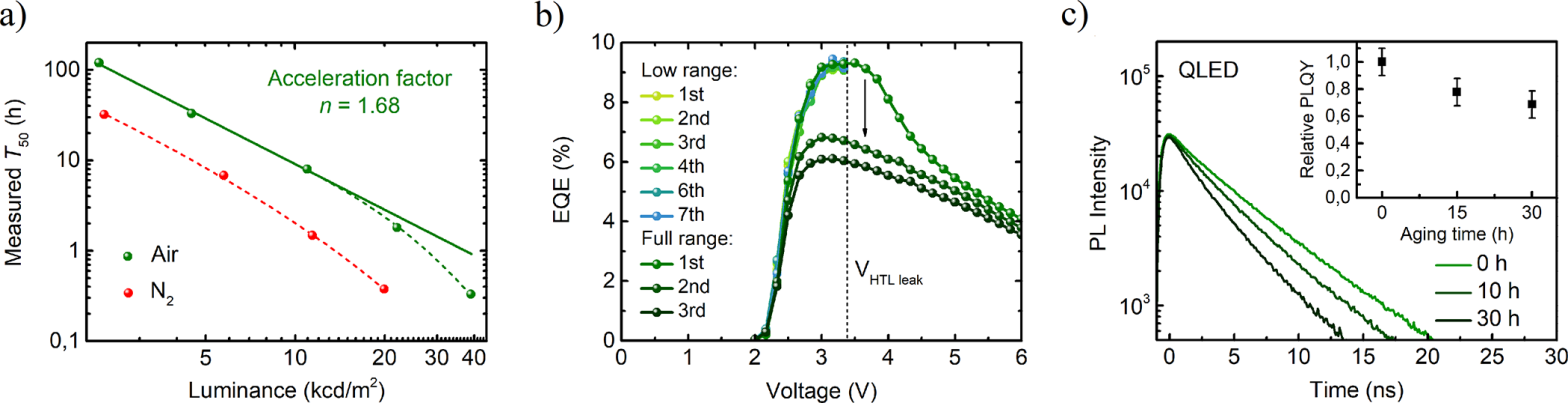
(a) QLED lifetime measured at different initial luminance for device with (Air) and without (N_2_) treatment of ZnMgO layer with moist air. (b) Reproducibility of EQE measurements at low range (0–3.3 V) and high range (0–6 V) for device treated with moist air. (c) Photoluminescence decays and relative PLQYs (inset) measured for the same device after aging the device at 3 V for a different time.

The improved charge balance of the device treated with air is additionally confirmed by much higher operational stability compared to the device without treatment (Figure 5a). However, the stability of the optimized device exhibits two different regimes. At a low voltage range (0–3.3 V), where the charge balance is high, accelerated aging satisfies the expression *L*_0_^*n*^LT_50_ = const, with the acceleration factor *n* ∼ 1.7. At higher voltage, a deviation of the LT_50_ value from the power law is observed instead. It was found that the EQE value is reproducible within the range of 0–3.3 V as long as the TFB layer is not electrochemically reduced, but it drops if the device is measured in the full range of 0–6 V (Figure 5b), which was also reported previously.^19^ This result shows that TFB degradation has a pronounced effect on device lifetime. After accelerated aging, the maximum EQE is reduced irreversibly because electron overflow aggravates TFB hole transport, which ruins the charge balance in the QDs layer. QDs also undergo irreversible degradation, which is evidenced by quenched PLQY and reduced exciton lifetime (Figure 5c). Electrochemical degradation is most likely related to poor stability of ligands,^38^ but other mechanisms including QDs etching by oxygen or PL quenching by trap states induced in TFB by electron leak are also possible.^14,24^

## Conclusions

In summary, we studied the influence of processing conditions on the optical and electrical properties of ZnMgO NPs, which allowed us to point out some of its important limitations. First, the air exposition of QLED is risky and requires precise control of exposure time and moisture level. Nevertheless, it does not have a detrimental effect on the optical properties of QDs and, more importantly, is beneficial to the device operation by reducing hole leakage. Second, adsorbed oxygen improves charge balance in QLED, introducing trap states which suppress electron transport in ZnMgO NPs. Apart from hole leakage, we also identified electron leakage into TFB as a third factor limiting device performance and shown that reduced electron injection helps to increase the leakage threshold. Although the sensitivity of ZnMgO NPs to oxygen and water has been taken as an advantage to boost EQE, it does not mean it is desirable in real applications. The obtained results leave, therefore, an open-ended question of whether ZnMgO is the optimal choice in terms of device stability and lifetime, which must be taken into consideration in the case of open-air manufacturing of QLEDs.

## Experimental Section

### Materials

Green-emitting CdSe@ZnS/ZnS QDs were synthesized according to the previously reported method.^39^ The hole transport materials TFB (poly(9,9-dioctylfluorene-*alt*-*N*-(4-*sec*-butylphenyl)diphenylamine), *M*_w_ ∼ 30000 g/mol) and TCTA (tris(4-carbazoyl-9-ylphenyl)amine) were delivered by Ossila Ltd. Materials for ZnMgO NPs synthesis including tetramethylammonium hydroxide pentahydrate (TMAH·5H_2_O, 97%), zinc acetate dihydrate (Zn(Ac)_2_·2H_2_O, >98%), magnesium acetate tetrahydrate (Mg(Ac)_2_·4H_2_O, >98%), DMSO (anhydrous, 99.9%), and ethyl acetate (anhydrous, 99.8%) were purchased from Sigma-Aldrich.

### ZnMgO NPs Synthesis

Colloidal Zn_0.85_Mg_0.15_O NPs were synthesized by a modified solution precipitation method.^40^ TMAH solution (2.5 mmol) in ethanol (4.5 mL) was added dropwise to the flask containing Zn(Ac)_2_·2H_2_O (1.28 mmol) and Mg(Ac)_2_·4H_2_O (0.23 mmol) dissolved in DMSO (15 mL). The solution was stirred for 1 h at room temperature under nitrogen flow. ZnMgO NPs were precipitated by using ethyl acetate and redispersed in anhydrous ethanol (99.9%) inside the glovebox.

### Device Fabrication

QLEDs with a structure of ITO/PEDOT:PSS/TFB:TCTA/QDs/ZnMgO/Al were fabricated in the nitrogen-filled glovebox. ITO substrates (20 Ω/sq) were rinsed in the ultrasonic cleaner and cleaned with detergent, isopropanol, and deionized water and then treated by UV-ozone cleaner for 15 min. PEDOT:PSS (Clevois AI 4083, filtered through 0.45 μm PES filter) was spin-coated at 4000 rpm for 45 s and baked at 130 °C for 15 min. TFB mixed with TCTA (10 mg/mL in chlorobenzene) was spin-coated at 4000 rpm and annealed at 120 °C for 20 min. QDs (10 mg/mL in octane) were deposited at 3000 rpm for 20 s. ZnMgO NPs (20 mg/mL in ethanol) were spin-coated at 4000 rpm and baked at 90 °C for 10 min. Devices were transferred to the high-vacuum sputter-coater and Al electrodes were deposited in Ar plasma with a rate of ∼20 Å/s at the pressure of 10^–2^ mbar. Finally, samples were encapsulated by using UV-curable resin (Pulse Puretone 20-001) and stored under N_2_.

### Characterization

ABS spectra of ZnMgO NPs were measured with Jasco V-550 UV/vis and AvaSpec-ULS2048XL spectrometers, and PL spectra were collected with an optical setup composed of 450 W xenon lamp, TRIAX 180 monochromator, and Ocean Optics HR4000 spectrometer. PLQY of QDs layers were measured in the integrating sphere (Gigahertz-Optic UPB-150-ART) coupled with AvaSpec-ULS2048XL spectrometer at 405 nm excitation. PL decays were measure at 520 nm utilizing time-correlated single-photon counting (TCSPC) setup composed of a 160 ps pulsed laser diode (450 nm, 1 MHz), a 480 nm long-pass edge filter, and a Horiba Jobin Yvon iHR 320 monochromator coupled with photon photomultiplier detector (PicoQuant PMA Hybrid 50). The thickness of QLED layers was determined from the depth of the scratch profile measured with a Park System XE-100 atomic force microscope working in tapping mode. FTIR spectra were recorded in transmission mode by a Nicolet iS10 spectrometer in dry conditions (RH < 10%) to prevent water adsorption. The XPS analysis was performed by using a SCIENTA R3000 hemispherical photoelectron spectrometer equipped with monochromatic Al Kα source operating at 300 W. The acquired spectra were calibrated to adventitious carbon at 284.8 eV. Device characterization was performed with a Konica Minolta LS-160 luminance meter coupled with a Keithley 2400 source-meter. For bias-dependent PLQY measurement, the pixel area was illuminated with frequency-modulated (200 Hz) excitation source (collimated LED), and the PL signal was detected with a Si photodiode and an EG&G Instruments 7265 DSP-based lock-in amplifier. To avoid direct excitation of the TFB layer, a 450 nm LED was selected. A Thorlabs FD1G dichroic filter was inserted in front of the photodetector to block scattered light. To ensure minimal impact of excitation on emission quenching, the PL signal was kept at an intensity corresponding to apparent luminance of 10 cd/m^2^. For QDs films embedded in unbiased devices, the relative PL signal was measured by illuminating the pixel area with a 450 nm LED and detecting luminance through a FD1G dichroic filter.

## Supporting Information Available

Optimization of *J–V–L* characteristics of QLED, *J–V* characteristics of QLED in moist air, PL decays of QDs thin films, the stability of QDs/ZnMgO, PL spectra of ZnMgO NPs, EL spectra of QLED, and PL spectra of TFB layer (PDF)
