# Supplementary material for: Effect of Air Exposure of ZnMgO Nanoparticle Electron Transport Layer on Efficiency of Quantum-Dot Light-Emitting Diodes
Source: ACS Appl Mater Interfaces. 2021 Apr 23;13(17):20305–12. doi: 10.1021/acsami.1c01898 (PMC8288913; doi:10.1021/acsami.1c01898)
Supplement: Supplementary file 1 — am1c01898_si_001.pdf [file am1c01898_si_001.pdf]

Supporting Information

Effect of air exposure of ZnMgO nanoparticle electron transport layer on efficiency of quantum-dot light-emitting diodes

*Maciej Chrzanowski, Grzegorz Zatryb, Piotr Sitarek, and Artur Podhorodecki**

Department of Experimental Physics, Wroclaw University of Science and Technology, Wybrzeze Wyspianskiego 27, 50-370 Wroclaw, Poland.

*E-mail: artur.p.podhorodecki@pwr.edu.pl

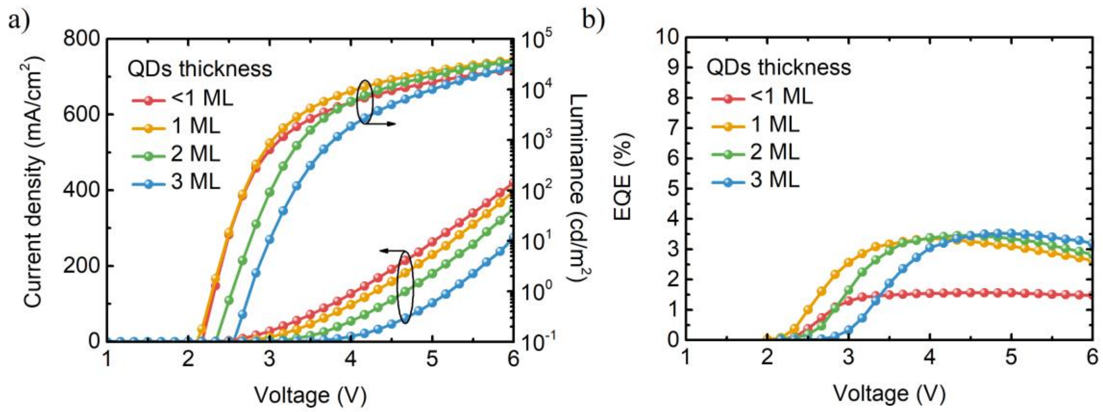

Figure S1. (a) *J–V–L* and (b) *EQE–J* characteristics of QLEDs with a different number of QDs monolayers.

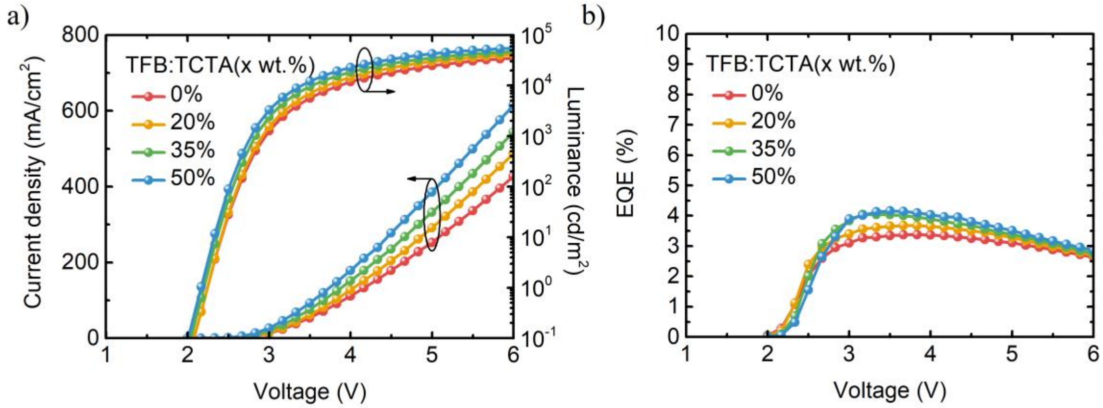

Figure S2. (a) *J–V–L* and (b) *EQE–J* characteristics of QLEDs with different HTL composition.

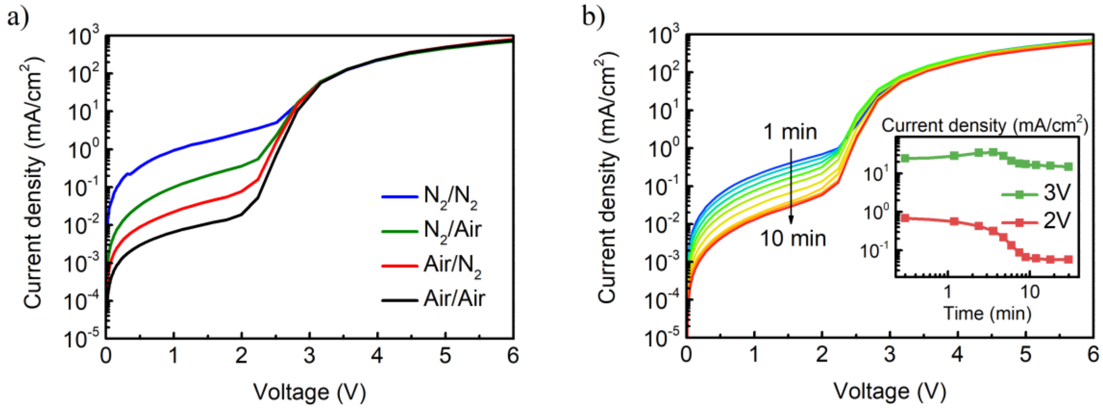

Figure S3. *J-V* characteristic of (a) QLEDs with ZnMgO exposed to air before or after Al deposition, and (b) fabricated in N_2 and exposed to air after Al deposition. Inset shows the evolution of a current density in ohmic and injection regimes.

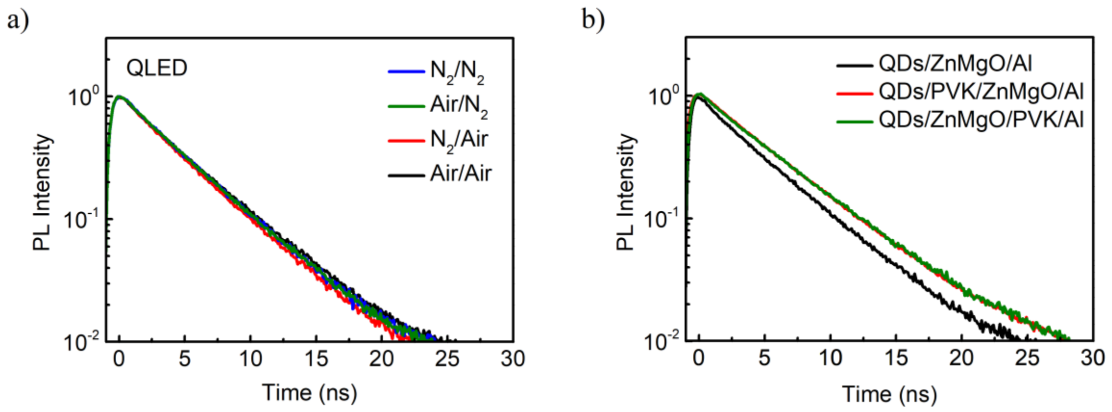

Figure S4. (a) PL decays measured from pixels of QLED with ZnMgO exposed to air in different sequences. (b) Effect of inserting 5 nm PVK buffer layer at QDs/ZnMgO or ZnMgO/Al interfaces on QDs exciton lifetime.

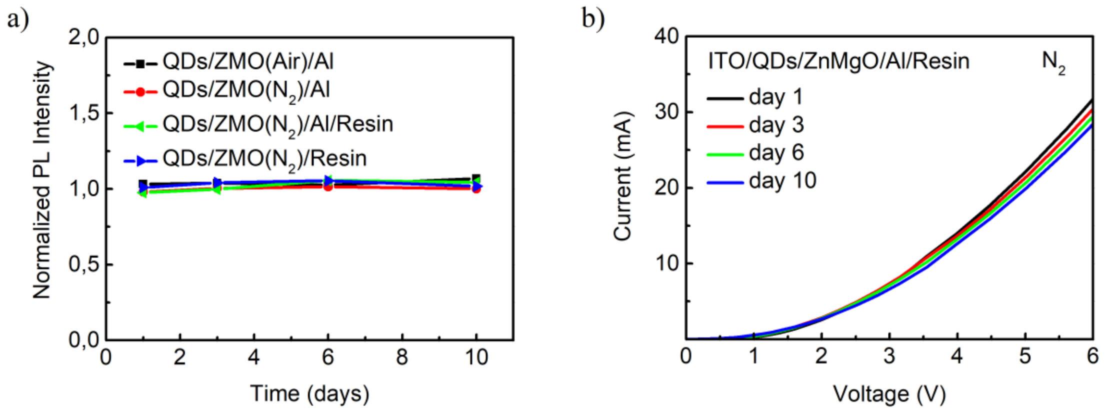

Figure S5. (a) Photoluminescence of QDs embedded with different configurations of ZnMgO NPs, Al electrode, and resin. (b) Reproducibility of electron current measurements in ZnMgO NPs films prepared in N_2.

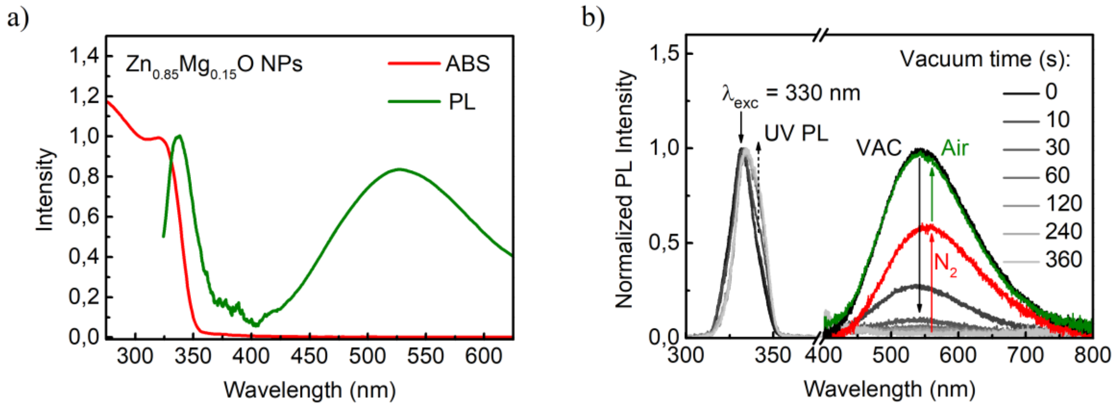

Figure S6. (a) Absorbance (ABS) and photoluminescence (PL) spectra of ZnMgO NPs dispersed in ethanol. (b) PL spectra of a thin film of ZnMgO NPs in a vacuum chamber at different time intervals after the pump is turned on (VAC) and after the chamber is vented successively with nitrogen (N_2) and air (Air).

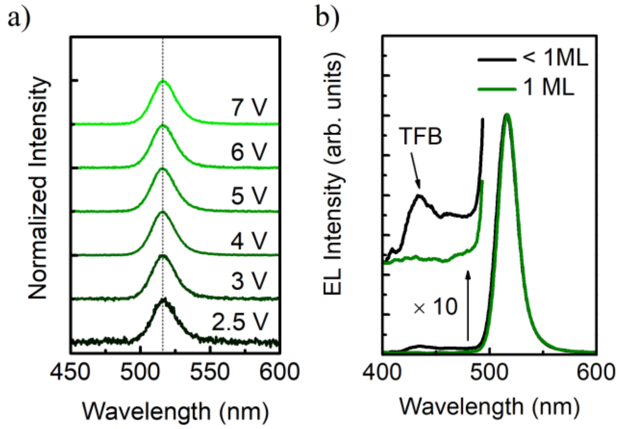

Figure S7. (a) EL spectra of the device with 1 ML-thick QDs layer measured under increasing bias. (b) Comparison of electroluminescence (EL) spectra of QLEDs with continuous (1 ML) and discontinuous (< 1 ML) QDs layer.

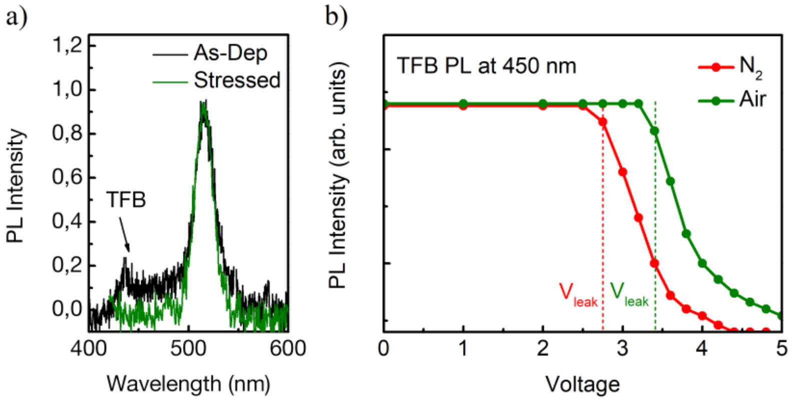

Figure S8. (a) Photoluminescence (PL) from the pixel area before and after the voltage is increased above the leakage threshold. (b) Voltage-dependent PL intensity of TFB layer for QLED with ZnMgO with and without air treatment.
